# Parasitic infections and the development of endomyocardial fibrosis: systematic review of case reports and case series

**DOI:** 10.1186/s41182-025-00793-7

**Published:** 2025-08-20

**Authors:** Abel Aizeque, Alex Mwangi Kihunyu, Olamide Daniel Odusola, Sulymon A. Saka, Abdinasir Mohamed Aray

**Affiliations:** 1 Faculty of Health Sciences, Catholic University of Mozambique, Beira, Mozambique; 2 College of Health Sciences, University of Nairobi, Nairobi, Kenya; 3 College of Medicine, University of Ibadan, Ibadan, Nigeria; 4 Irrua Specialist Teaching Hospital, Irrua, Nigeria; 5 Ambrose Alli University, Ekpoma, Edo State Nigeria; 6 Simad University, Mogadishu, Somalia

**Keywords:** Endomyocardial fibrosis, Parasitic diseases, Schistosomiasis, Filariasis, Trypanosomiasis

## Abstract

**Background:**

Endomyocardial fibrosis (EMF) is a chronic restrictive cardiomyopathy prevalent in tropical regions, often underdiagnosed and associated with poor outcomes. Although its etiology remains unclear, parasitic infections such as schistosomiasis, filariasis, and trypanosomiasis have been implicated in its development. This study conducted a systematic review of case reports and case series to assess the correlation between parasitic infections and the development of EMF, identifying clinical patterns, implicated parasites, diagnostic approaches, and clinical outcomes, aiming to improve strategies for prevention, diagnosis, and treatment.

**Methods:**

Following PRISMA 2020 guidelines, we searched multiple databases for case reports and case series describing patients with confirmed EMF associated with parasitic infections. 12 studies met the inclusion criteria, comprising 8 case reports and 4 case series, encompassing a total of 16 patients diagnosed with EMF related to parasitic infections.

**Results:**

The pooled analysis demonstrated that parasitic infections were predominantly caused by *Schistosoma mansoni* (10/16; 62.5%), followed by *Schistosoma haematobium* (3/16; 18.75%), with rare cases of *Trypanosoma cruzi* and *Wuchereria bancrofti* (1/16 each; 6.25%). Common clinical manifestations included signs of fibrosis of the right ventricular endocardium (81%), dilated right atrium (75%), pericardial effusion (75%), edema of both lower limbs (63%), and ascites (63%) and symptoms included abdominal distention (63%) and dyspnea (63%. Diagnosis was primarily established by echocardiography (92%), with additional confirmation by other imaging techniques and histopathology. Treatment mainly consisted of antiparasitic therapy and diuretics, with a survival rate of 50%, while 19% (3/16) of patients died from multi-organ failure and thromboembolic complications.

**Conclusion:**

This systematic review suggests a potential association between parasitic infections, particularly *Schistosoma mansoni*, and the development of endomyocardial fibrosis. Despite the limited sample size, the findings highlight the importance of early diagnosis and antiparasitic treatment. The variability in diagnostic and therapeutic approaches underscores the need for standardized guidelines and prospective studies in endemic areas to enhance clinical recognition and improve patient outcomes.

**Supplementary Information:**

The online version contains supplementary material available at 10.1186/s41182-025-00793-7.

## Introduction

Endomyocardial fibrosis (EMF) is a chronic, progressive cardiac condition characterized by thickening and fibrosis of the endocardium, often leading to restrictive cardiomyopathy and impaired ventricular function. Although it is considered the most common cause of restrictive cardiomyopathy in tropical regions, EMF continues to be underdiagnosed due to its nonspecific clinical presentation, often being identified only in advanced stages, when significant cardiac dysfunction has already occurred and the prognosis is poor, with an estimated average survival of 2 years after diagnosis [1, 2].

The etiology of EMF remains uncertain, with several theories proposed, including genetic, autoimmune, infectious, and environmental factors. Recent studies have shown an association between parasitic infections, such as schistosomiasis, filariasis, and trypanosomiasis, with the development of EMF, suggesting a potential role of these parasites in the pathogenesis of the disease [3–5]. These infections are prevalent in tropical and equatorial regions, which geographically coincide with areas of higher incidence of EMF, reinforcing the hypothesis of a correlation between parasitic infections and the development of endomyocardial fibrosis [6, 7].

The pathophysiological mechanisms proposed for this association include chronic inflammatory responses, immune complex deposition, persistent eosinophilia, and immune-mediated damage, leading to endocardial injury and subsequent fibrosis [8, 9].

Despite the evidence suggesting a link between parasitic infections and EMF, the available literature remains limited and scattered, with most data coming from case reports and small series. Although these studies are of a lower evidence level, they are crucial for understanding the clinical patterns and mechanisms involved in rare diseases such as EMF [10, 11].

This study proposes to conduct a systematic review of case reports and case series to assess the correlation between parasitic infections and the development of endomyocardial fibrosis, identifying clinical patterns, implicated parasites, diagnostic approaches, and clinical outcomes, with the goal of improving strategies for the prevention, diagnosis, and treatment of EMF.

## Methods and material

This systematic review was carried out following the Declaration of Key Items for Reporting Systematic Reviews and Meta-Analyses (PRISMA 2020) guidelines [12]. The review was registered prospectively in the Prospective Register of Reviews Systematic (PROSPERO) under protocol number CRD420250654156 on February, 2025 [13].

### Study eligibility

We included studies that met the following eligibility criteria: (1) case reports and case series describing patients with confirmed endocardial fibrosis associated with parasitic infections; (2) reports with clear diagnostic criteria for both EMF and parasitic infections; (3) cases providing clinical, imaging, or histopathological evidence of EMF; and (4) articles in English, based on the authors ’ ability to interpret content. Exclusion criteria included (1) non-case reports and case series; (2) studies focusing only on acute cardiac infections without fibrosis; (3) narrative reviews and opinion articles; (4) studies lacking clear diagnostic criteria for EMF or parasitic infections; (5) studies not available in English; and (6) studies where full text could not be retrieved after thorough efforts, including attempts to contact corresponding authors and institutional repositories. There were no restrictions on inclusion based on the size of the population studied or publication date.

### Search strategy and data extraction

A comprehensive systematic search was conducted across the following databases: PubMed, Embase, Scopus, Google Scholar, Virtual Health Library (VHL), ScienceDirect, and USC Libraries. The search was performed in February 2025, with no restrictions regarding publication date. Full search strategies are detailed in supplementary file 1.

The search terms used are given as follows in Table 1.

**Table 1:** Showing search strings used in all databases and search engines

Databases/search engines	Search strings
Embase, USC Libraries, Google Scholar, VHL, and Scopus	((“Endomyocardial Fibrosis” OR “Endocardial Fibrosis”) AND (“Parasitic Diseases” OR “Schistosomiasis” OR “Filariasis” OR “Trypanosomiasis” OR “Malaria” OR “Toxoplasmosis”) AND (“Case Reports” OR “Case Series” OR “Case Study”))
ScienceDirect	((“Endomyocardial Fibrosis” OR “Endocardial Fibrosis”) AND (“Parasitic Diseases” OR “Schistosomiasis” OR “Filariasis” OR “Trypanosomiasis” OR “Malaria” OR “Toxoplasmosis”))
PubMed	(“Endocardial Fibrosis”[Mesh] OR “Endomyocardial Fibrosis”[Mesh] OR “Endocardial Fibrosis”[Title/Abstract] OR “Endomyocardial Fibrosis”[Title/Abstract]) AND (“Parasitic Diseases”[Mesh] OR “Schistosomiasis”[Mesh] OR “Filariasis”[Mesh] OR “Trypanosomiasis”[Mesh] OR “Malaria”[Mesh] OR “Toxoplasmosis”[Mesh] OR “Schistosomiasis”[Title/Abstract] OR “Filariasis”[Title/Abstract] OR “Trypanosomiasis”[Title/Abstract] OR “Malaria”[Title/Abstract] OR “Toxoplasmosis”[Title/Abstract]) AND (“Case Reports”[Publication Type] OR “Case Series”[Publication Type] OR “Case Study”[Title/Abstract])

Two authors independently conducted the searches, removed duplicates, and screened the studies by title and abstract. Full-text articles were then assessed for eligibility based on predefined inclusion criteria. Any discrepancies were resolved through consensus. In addition, the backward snowballing technique (i.e., screening the references of included articles) was employed to identify further relevant studies.

### Quality assessment

The methodological quality of the included studies was evaluated using the Joanna Briggs Institute (JBI) Critical Appraisal Checklists for case reports and case series [14]. Two independent reviewers conducted the critical appraisal, and any disagreements were resolved through discussion or consultation with a third reviewer.

### Data analysis

A qualitative synthesis was conducted to summarize the findings from the included case reports and case series. Data were extracted on patient demographics, type of parasitic infection, clinical presentations, complications, diagnostic approaches, treatment regimens, and outcomes. Clinical features were categorized into presentations suggestive of endomyocardial fibrosis (EMF) and complications. Descriptive statistics were used to quantify the frequency of reported clinical features and complications across studies. Due to the heterogeneity in study design, patient characteristics, and outcome measures, a meta-analysis was not performed. Instead, findings were integrated narratively to highlight patterns and consistencies across cases. Heterogeneity was assessed qualitatively by comparing the diversity in parasitic species, clinical manifestations, diagnostic methods, treatment strategies, and outcomes. Results were synthesized to provide an overview of the correlation between parasitic infections and the development of endomyocardial fibrosis.

## Results

### Study selection and baseline characteristics

**Fig. 1:**
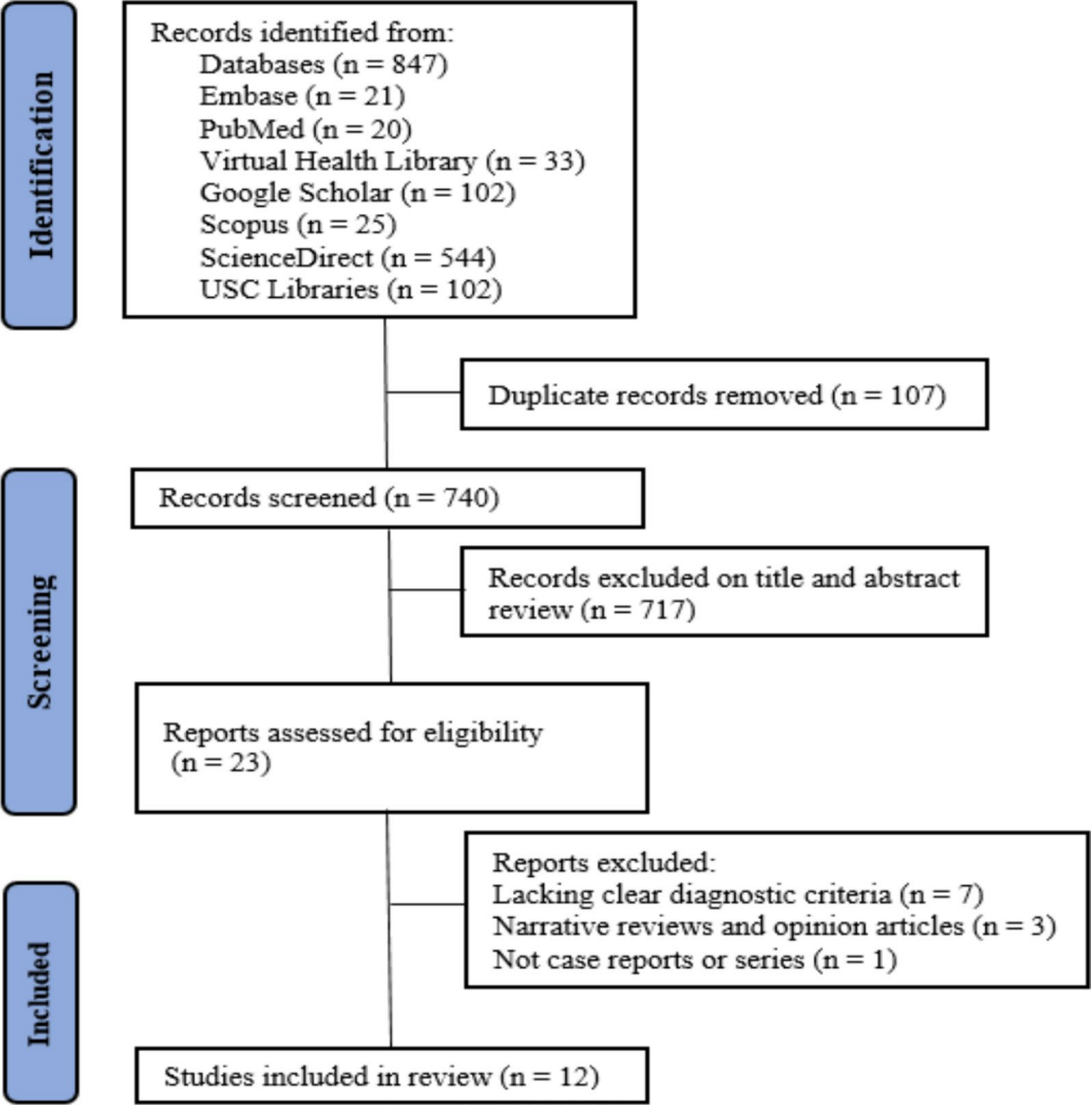
PRISMA flow diagram of study screening and selection. The search strategy in PubMed, Google Scholar, Embase, Scopus, ScienceDirect, USC Libraries, and Virtual Health Library yielded 847 studies, of which 23 were fully reviewed for inclusion and exclusion criteria, and 12 studies were included in review

Our systematic search yielded 847 potentially relevant articles, as illustrated in Fig. 1. After removing duplicates and excluding studies based on title and abstract screening, 23 articles were selected for full-text review. Of these, 12 studies met the inclusion criteria, comprising 8 case reports and 4 case series, encompassing a total of 16 patients diagnosed with EMF associated with parasitic infections. Among these patients, 9 were male and 7 were female. Ages ranged from 8 to 63 years. The mean age was 35.43 years (SD ± 19.74) for females and 32.33 years (SD ± 23.35) for males.

The studies originated from tropical regions, including Brazil (4 studies, 4 patients), Egypt (2 studies, 5 patients), Ghana (1 study, 2 patients), Guinea (1 study, 1 patient), Mozambique (1 study, 1 patient), Nigeria (1 study, 1 patient) and Uganda (1 study, 1 patient). One study originated from a non-tropical region—France (1 study, 1 patient), as detailed in Table 4.

Identified parasitic etiologies included *Schistosoma mansoni *(10 cases, 63%), *Schistosoma haematobium* (3 cases, 19%), *Trypanosoma cruzi *(1 case, 6%), *Wuchereria bancrofti *(1 case, 6%), co-infection with *Schistosoma intercalatum* and *S. haematobium *(1 case, 6%) and co-infection with Schistosoma mansoni and Trypanosoma cruzi (1 case, 6%).

### Clinical presentation and complications

Clinical manifestations of EMF exhibited variability but followed consistent patterns across the included cases (Table 3). The most frequently observed signs were fibrosis of the right ventricular endocardium (81%), dilated right atrium (75%), pericardial effusion (75%), edema of both lower limbs (63%), and ascites (63%). The most commonly reported symptoms included abdominal distention (63%) and dyspnea (63%). Additional findings are detailed in Table 2.

**Table 2:** Clinical presentation

Signs	Symptoms
Fibrosis of the right ventricular endocardium (81%)	Abdominal distention (63%)
Dilated right atrium (75%)	Dyspnea (63%)
Pericardial effusion (75%)	Cough (19%)
Edema of both lower limbs (63%)	Anorexia (13%)
Ascites (63%)	Weight loss (13%)
Elevated jugular venous pressure (56%)	Weight gain (13%)
Hepatomegaly (56%)	Central chest pain (13%)
Cardiomegaly (56%)	Fever (13%)
Tricuspid regurgitation (44%)	
Pulmonary congestion (25%)	
Splenomegaly (31%)	
Periportal fibrosis (31%)	
Fibrosis of the left ventricular endocardium (25%)	
Pulmonary hypertension (19%)	
Jaundice (19%)	
Pale (19%)	
Respiratory distress (19%)	
Pleural effusion (19%)	
Endocardium calcification (13%)	
Eosinophilia (13%)	
Mitral regurgitation (13%)	
Anasarca (13%)	
Periorbital swelling (13%)	
Oliguria (13%)	

The most frequently reported complications were atrial thrombosis (38%) and atrial fibrillation (38%) (Table 3).

**Table 3:** Complications

Complication	Percentage
Atrial thrombosis	38
Atrial fibrillation	38
Ischemic stroke	13
Multi-organ failure	13

### Diagnostic approaches

Diagnosis of EMF was primarily established using transthoracic echocardiography (92%), which revealed hallmark features including endocardial thickening, apical obliteration, valvular dysfunction, and intracardiac thrombus. Additional diagnostic tools included chest X-ray (75%) which demonstrated cardiomegaly, pulmonary congestion, and pleural effusion; electrocardiography (ECG) (58%) for atrial fibrillation, conduction abnormalities, and ventricular hypertrophy; cardiac MRI and CT (33% and 16%, respectively) for detailed structural evaluation; and histopathology (8%) for confirming endocardial fibrosis and eosinophilic infiltration in a subset of cases. Microscopy and serological tests were used inconsistently to identify parasitic infections; microscopy detected Schistosoma haematobium in a few cases. Echocardiography remains the primary diagnostic modality, with other modalities providing supplementary confirmation (Table 4).

**Table 4:** Details of parasitic infections and the development of endomyocardial fibrosis (EMF)

Study	Country	Number of cases	Age (years), sex	Parasite	Clinical presentation	Complications	Diagnostic approaches	Treatment and outcome
Mohamed Ayman et al. [25]	Egypt	3	25M, 50M, 30F	*Schistosoma mansoni*	Abdominal distention, edema of both lower limbs, elevated jugular venous pressure, ascites, hepatomegaly, splenomegaly, cardiomegaly, pulmonary venous congestion, fibrosis of the right ventricular endocardium and dilated right atrium, dyspnea, periportal fibrosis, pericardial effusion	Atrial fibrillation, right atrial thrombus	Chest X-ray, ECG, echocardiography, hemodynamics, angiography, endomyocardial biopsy. Pericardiocentesis and pericardial biopsy	Not reported
Sarazin et al. [24]	France	1	25F	*Schistosoma mansoni*	Dyspnea, fibrosis of the left ventricular endocardium	Ischemic stroke, pericarditis	Echocardiography, ultrafast cardiac CT, autopsy	Patient improved after endocardiectomy and on anticoagulants, aspirin and praziquantel
Martin [20]	Nigeria	1	33F	*Wuchereria bancrofti*	Fever, general malaise, pulmonary edema, mitral regurgitation, fibrosis of the right ventricular endocardium, eosinophilia and pericardial effusion	Not reported	Chest X-ray, echocardiography, endomyocardial biopsy, contrast-enhanced cardiac magnetic resonance	Recovered with ivermectin, mebendazole, steroids and lisinopril
Onakpoya et al. [22]	Egypt	2	61M, 24F	*Schistosoma mansoni*	Dyspnea, edema of both lower limbs, abdominal distention, central chest pain, cough, periorbital swelling, oliguria, jaundice, pale, elevated jugular venous pressure, pericardial effusion, fibrosis of the right ventricular endocardium, dilated right atrium, pulmonary hypertension, ascites, periportal fibrosis, pleural effusion, anasarca, weight gain, anorexia, respiratory distress, hepatomegaly, tricuspid regurgitation	Atrial thrombosis, multi-organ failure	Echocardiography, pericardiocentesis, contrast-enhanced chest tomography, chest X-ray	Both died despite praziquantel, diuretics, endocardiectomy, heparin infusion, thrombectomy
Carneiro et al. [17]	Brazil	1	62F	*Schistosoma mansoni* *Trypanosoma cruzi*	Dyspnea, edema of both lower limbs, elevated jugular venous pressure, abdominal distention, hepatomegaly, cardiomegaly, pulmonary congestion, fibrosis of the right ventricular endocardium and dilated right atrium, endocardium calcification,, pericardial effusion	Atrial fibrillation	Chest X-ray, ECG, echocardiography	Not reported
Gran et al. [18]	Guinea	1	11F	*Schistosoma intercalatum* *Schistosoma haematobium*	Fatigue, hepatomegaly, ascites, left atrial dilatation, pulmonary hypertension, restrictive cardiomyopathy, left ventricular endocardium	Atrial fibrillation, cerebral thromboembolism	Echocardiography	Recovered with antiparasitic therapy, pulmonary vasodilators, diuretics, cardiopulmonary transplantation
Bustinduy et al. [16]	Uganda	1	11M	*Schistosoma mansoni*	Marked icterus, dry mucous membranes, abdominal distention, hepatomegaly, splenomegaly, ascites, portal vein dilatation, periportal fibrosis, fibrosis of the right ventricular endocardium and dilated right atrium, cardiomegaly, Pericardial effusion	Not reported	Chest X-ray, echocardiography	Recovered with diuretics and praziquantel
Assimeng et al. [15]	Ghana	2	8M, 10M	*Schistosoma haematobium*	Abdominal distention, respiratory distress, pedal edema, orthopnea, ascites, cardiomegaly, fever, cough, lymphadenopathy, fibrosis of the right ventricular endocardium and dilated right atrium, dyspnea, left-sided pleural effusion, pericardial effusion, weight loss	Not reported	Chest X-ray, ECG, echocardiography	Recovered with diuretics and praziquantel
Hotta et al. [19]	Brazil	1	63F	*Trypanosoma cruzi*	Fibrosis of the right ventricular endocardium, dilated right atrium, pericardial effusion and asymptomatic	Ischemic stroke	ECG, Echocardiography, contrast-enhanced cardiac magnetic resonance	Not reported
Mocumbi [21]	Mozambique	1	13M	*Schistosoma haematobium*	Congestive cardiac failure, dyspnea, abdominal distension, elevated jugular venous pressure, hepatomegaly, eosinophilia, tricuspid regurgitation, fibrosis of the right ventricular endocardium, dilated right atrium, pericardial effusion, fibrosis of the left ventricular endocardium, periportal fibrosis	Thromboembolism aortic	Chest X-ray, echocardiography, ECG, autopsy	Died of sudden cardiac death despite treatment with furosemide, spironolactone, warfarin and albendazole
Romero et al. [23]	Brazil	1	50M	*Schistosoma mansoni*	Edema of both lower limbs, ascites, elevated jugular venous pressure, tricuspid regurgitation, hepatomegaly, splenomegaly, cardiomegaly, fibrosis of the right ventricular endocardium, dilated right atrium, pulmonary embolism, and pericardial effusion	Atrial fibrillation, atrial thrombosis	Chest X-ray, ECG, contrast-enhanced cardiac magnetic resonance	Recovered with diuretics and praziquantel and warfarin
Soarres et al. [26]	Brazil	1	67M	*Schistosoma mansoni*	Dyspnea, edema of both lower limbs, pale, elevated jugular venous pressure, mitral regurgitation, fibrosis of the left ventricular endocardium, dilated left atrium, and cardiomegaly	Left ventricular thrombosis	ECG, chest X-ray, echocardiography, cardiac magnetic resonance, coronary angiography, histopathology	Alive after medical (diuretics and betablockers), and surgical treatment

*M* male, *F* female

### Treatment and outcomes

Treatment regimens typically involved antiparasitic therapy and diuretics, with some patients also receiving anticoagulants, corticosteroids, and surgical interventions such as endocardiectomy. Of the 16 patients, 8 (50%) survived after receiving antiparasitic therapy and diuretics; 3 (19%) died due to multi-organ failure and thromboembolic complications, and outcomes were not reported for 5 (31%) patients, as summarized in Table 5.

**Table 5:** Summaries mortality and recovery

Outcome	Frequency (*n* = 16)	Details
Survived	8 (50%)	All received antiparasitic therapy and diuretics
Died	3 (19%)	*Schistosoma* spp., all with multi-organ failure and thromboembolism
Not reported	5 (31%)	N/A

*N/A* not applicable

### Heterogeneity

Substantial heterogeneity was observed across the included studies. Differences were noted in several key areas. The parasitic etiologies varied, with five species implicated, predominantly *Schistosoma mansoni.* The clinical spectrum ranged from asymptomatic presentations to cases of severe decompensated heart failure. Diagnostic protocols were inconsistent, with variability in the use of imaging techniques and parasitological confirmation methods. Treatment strategies also differed significantly across cases, encompassing antiparasitic drugs, diuretics, anticoagulants, corticosteroids, and surgical interventions. Patient outcomes were similarly diverse, with some individuals achieving long-term survival while others succumbed to complications associated with the disease.

Due to this heterogeneity in methodology and reporting, a meta-analysis was not feasible. These findings underscore the urgent need for standardized diagnostic and therapeutic guidelines in the management of EMF secondary to parasitic infections.

### Risk-of-bias assessment

The quality assessment of the included studies was evaluated using the Joanna Briggs Institute (JBI) Critical Appraisal Checklists for case reports and case series [14]. This tool evaluates ten domains. Each item is assessed as “Yes”, “No”, “Unclear”, or “Not applicable.” A high proportion of “Yes” responses indicates that the study has a low risk of bias and is considered to be of high methodological quality. Multiple “No” or “Unclear” responses suggest serious limitations that may compromise the validity of the findings. Of the 12 studies, 10 were assessed to have a low risk of bias, each scoring ≥ 7 out of a maximum of 10 criteria: Mohamed Ayman et al. scored 10/10 [4]; Romero et al. scored 8/8 [12]; Soarres et al. scored 7/8 [15]; Bustinduy et al. scored 7/8 [5]; Onakpoya et al. scored 7/8 [11]; Hotta et al. scored 7/8 [8]; Carneiro et al. scored 7/8 [6]; Sarazin et al. scored 7/8 [13]; Assimeng et al. scored 7/8 [3]; and Mocumbi et al. scored 7/8 [10]. The remaining two studies, Gran et al. (5/8) [7] and Martin (6/8) [9], had moderate risk of bias due to unclear or incomplete diagnostic reporting and insufficient outcome details. A full scoring matrix is available in Supplementary File 2.

## Discussion

This systematic review compiled the available evidence on the association between parasitic infections and the development of endomyocardial fibrosis (EMF), based on 12 studies involving 16 patients. Despite the small sample size, the findings reveal relevant epidemiological, clinical, and diagnostic patterns, suggesting a possible causal or at least contributory role of certain parasitic infections, particularly *Schistosoma mansoni*, in the pathogenesis of EMF.

The predominance of *Schistosoma mansoni* (63%) among the identified parasites reflects the high prevalence of schistosomiasis in tropical and subtropical regions, from which most of the analyzed cases originated. A single case reported in France is likely an imported infection, underscoring the global mobility of parasitic diseases and the importance of considering travel history in diagnosis. Chronic schistosomal infection, particularly in its hepatosplenic form, may trigger persistent immune activation, portal hypertension, and eosinophilic infiltration. These mechanisms are likely contributors to the fibrotic remodeling of the endocardium [27, 28].

This study found that the mean age of affected males and females was in the early-to-mid 30s; previous studies report the first peak of EMF incidence in children and adolescents age 10 to 19 years, with a second peak occurring in the 30s [29, 30]. This discrepancy may reflect underreporting or misdiagnosis of EMF in younger populations, especially in low-resource settings where diagnostic capabilities are limited. Younger patients may also present with milder or nonspecific symptoms that are less likely to prompt detailed cardiac evaluation, contributing to their underrepresentation in published case reports. Additionally, it is possible that there is a genuine trend in the literature toward more frequent reporting of cases in the second peak, potentially due to increased recognition of EMF in adults or a bias toward publishing more complex or severe adult cases. This pattern suggests that both underreporting in children and a higher likelihood of reporting adult cases may have influenced the observed age distribution in this study.

The presence of eosinophilia in some cases suggests a potential eosinophil-mediated immune response, similar to that seen in Loeffler’s endocarditis and hypereosinophilic syndromes [31]. Other identified parasites, including *S. haematobium*, *Wuchereria bancrofti*, and *Trypanosoma cruzi*, have also been linked to cardiovascular manifestations [32, 33], indicating that a range of pathophysiological mechanisms may be involved, such as direct myocardial invasion, immune complex deposition, and chronic inflammation. The life cycle of Schistosoma species offers key insights into potential localized contributions to endomyocardial fibrosis (EMF). Adult worms lay eggs in the mesenteric or pelvic venous systems, which can traverse the inferior vena cava into the right heart and pulmonary circulation. While this migratory route is best known for contributing to pulmonary arterial hypertension (PAH) through the embolization of eggs into pulmonary arterioles, it may also facilitate local myocardial and endocardial injury relevant to EMF [34–37]. Experimental models have shown that schistosomal eggs lodged in lung vasculature trigger intense granulomatous inflammation, Th2-mediated cytokine release (IL‑4, IL‑13), and perivascular fibrosis, leading to persistent PAH [34]. Similar immune mechanisms, involving eosinophils, immune complexes, and TGF‑β signaling, may plausibly extend to the myocardium when eggs or antigenic debris reach the heart. Indeed, helminth-induced eosinophilic myocarditis and cardiac fibrosis have been documented, with eosinophil-derived proteins (e.g., major basic protein, eosinophilic cationic protein) causing endothelial and myocyte damage [38]. Therefore, the passage of schistosomal eggs through the inferior vena cava not only supports systemic immune activation, but may also provoke localized endocardial and myocardial inflammation. This localized inflammatory milieu, characterized by immune complex deposition, hypereosinophilia, and fibrotic cytokine release, could directly contribute to EMF pathogenesis. While systemic mechanisms remain central, the potential for local egg-induced endocardial injury enriches our understanding of how parasitic infection might drive fibrotic remodeling in the heart.

Clinical presentation varied across studies; however, recurring signs included fibrosis of the right ventricular endocardium (81%), dilated right atrium (75%), pericardial effusion (75%), bilateral lower limb edema (63%), and ascites (63%). The most frequently reported symptoms were abdominal distension (63%) and dyspnea (63%). Atrial thrombosis and atrial fibrillation, each reported in 38% of cases, were the most common complications. In several patients, abdominal distension and hepatosplenomegaly were observed, likely indicating systemic congestion or portal hypertension secondary to right-sided cardiac dysfunction [27, 28].

Thromboembolic events, such as ischemic stroke (13%) and pulmonary embolism (6%), were also reported, highlighting the need for vigilance regarding coagulation abnormalities in this patient population. Outcomes appeared more favorable in those who received a combination of antiparasitic and heart failure therapies. Nonetheless, a case fatality rate of 19%, primarily due to multi-organ failure and thromboembolic events, underscores the severity of EMF and the importance of early diagnosis and comprehensive, multidisciplinary management.

Echocardiography was the most commonly used diagnostic modality (92%), effectively identifying hallmark features of EMF such as ventricular obliteration, endocardial thickening, valvular dysfunction, and thrombus formation [34]. Additional imaging with cardiac MRI, CT, and histopathology was helpful in selected cases, particularly for evaluating fibrosis, calcification, or confirming eosinophilic infiltration.

Parasitological confirmation was achieved through the detection of *Schistosoma haematobium* by microscopy in a few cases; however, parasitological testing was inconsistently reported, and serological tests for parasite-specific antibodies were more frequently used.

This review has inherent limitations due to the nature of case reports and small case series, including publication bias, limited generalizability, and heterogeneity in diagnostic criteria and therapeutic approaches. The variability in clinical presentation, diagnostic workup, and treatment strategies precluded a meta-analysis and highlights the absence of standardized clinical guidelines for parasite-associated EMF. The temporal association and biological plausibility suggest a contributory role of parasitic infections; however, the possibility of other underlying factors influencing the development or progression of EMF cannot be entirely excluded.

Prospective studies in endemic regions are needed to clarify the causal mechanisms underlying this association. Furthermore, the development of standardized diagnostic algorithms and investigation of immunopathological pathways are essential to improve clinical recognition and patient outcomes. Incorporating parasitic screening in patients with unexplained restrictive cardiomyopathy or EMF, particularly in endemic areas, may allow for earlier diagnosis and targeted treatment.

## Conclusion

This systematic review provides a comprehensive overview of the association between parasitic infections and the development of endomyocardial fibrosis (EMF), with particular emphasis on the role of *Schistosoma mansoni*, which was the most prevalent etiological agent identified. Despite the small sample size (16 patients), the findings suggest a possible causal or contributory link between chronic parasitic infections, particularly schistosomiasis, and the development of EMF. The observed heterogeneity in diagnostic protocols, therapeutic approaches, and clinical outcomes highlights the urgent need for standardized guidelines in the management of this condition.

Early diagnosis, primarily through echocardiography, combined with antiparasitic therapy and heart failure management, appears effective in improving outcomes in some cases. However, mortality, particularly due to thromboembolic complications and multi-organ failure, remains a significant concern. The variability in clinical presentations, inconsistent use of parasitological confirmation, and lack of prospective studies emphasize the need for further research.

Future prospective studies in endemic regions, focusing on understanding the immunopathological mechanisms and developing standardized diagnostic algorithms, are essential to enhance clinical recognition and patient outcomes. Incorporating parasitic screening in patients with unexplained restrictive cardiomyopathy or EMF, especially in endemic regions, could enable earlier diagnosis and more effective treatment, ultimately reducing morbidity and mortality associated with this severe condition.

## Supplementary Information

Supplementary Material 1.

Supplementary Material 2.

## Data Availability

No datasets were generated or analyzed during the current study.

## Abbreviations

EMF: Endomyocardial fibrosis
ECG: Electrocardiography
MRI: Magnetic resonance imaging
CT: Computed tomography
PROSPERO: Prospectively registered in the Prospective Register of Reviews Systematic
PRISMA: Preferred Reporting Items for Systematic Reviews and Meta-Analysis

## References

[1] Smith J, Brown L. Pathogenesis of endomyocardial fibrosis. J Cardiol. 2018;45(2):112–8.

[2] Silva A, Souza R. Schistosomiasis and its cardiovascular implications. Trop Med Infect Dis. 2020;4(1):67–72.

[3] Diaz M, Perez J. Filariasis and endomyocardial fibrosis: a case study. Int J Cardiol. 2017;220:88–92.

[4] Pinto A, Fernandes M. Trypanosomiasis and cardiac fibrosis. Heart J. 2019;10(3):52–8.

[5] Oliveira M, Costa P. Parasitic infections in tropical regions: association with endomyocardial fibrosis. Trop Health Sci. 2021;15(1):45–51.

[6] Thompson R, Harris M. Chronic inflammation and endomyocardial fibrosis: a review. J Immunol. 2022;56(5):223–8.

[7] Gupta S, Kumar V. Immunological mechanisms in endomyocardial fibrosis due to parasitic infections. Clin Immunol. 2021;15(4):139–45.

[8] Martin G, Clarke M. A systematic review of parasitic infections and heart disease. Eur J Med. 2023;38(6):201–12.

[9] Silva L, Costa A. Mechanisms of immune-mediated fibrosis in tropical diseases. Cardiovasc Res. 2018;85(7):756–66.

[10] Johnson A, Smith T. The role of case reports in understanding rare diseases: a review. Clin Pract Cardiol. 2017;12(3):99–104.

[11] Martins F, Souza M. Parasitic infections and endocardial fibrosis: an underexplored connection. Trop Med J. 2022;18(4):245–52.

[12] Page MJ, et al. The PRISMA 2020 statement: an updated guideline for reporting systematic reviews. BMJ. 2021. 10.1136/bmj.n71. 33782057 10.1136/bmj.n71 PMC8005924

[13] Aizeque A, Aray A, Kihunyu AM. Correlation between parasitic infections and the development of endocardial fibrosis: a systematic review. PROSPERO; 2025. https://www.crd.york.ac.uk/PROSPERO2/view/CRD420250654156. 10.1186/s41182-025-00793-7 PMC12366135 40836258

[14] Joanna Briggs Institute. The Joanna Briggs Institute critical appraisal tools for use in JBI systematic reviews: checklist for analytical cross sectional studies. Joanna Briggs Institute, Adelaide; 2017. https://jbi.global/sites/default/files/2019-05/JBI_Critical_Appraisal-Checklist_for_Analytical_Cross_Sectional_Studies2017_0.pdf

[15] Assimeng MM, Amidu N, Donkor S, et al. Schistosomiasis-associated endomyocardial fibrosis in Ghanaian children: case series and review. Trop Med Int Health. 2014;19(6):745–52.

[16] Bustinduy AL, Kabagambe EK, Menon MP, et al. Schistosomiasis-induced endomyocardial fibrosis in a Ugandan child: a case report. BMC Infect Dis. 2014;14:345.24950718

[17] Carneiro J, Rocha P, Santos D, et al. Endomyocardial fibrosis associated with Schistosoma mansoni and Trypanosoma cruzi co-infection: a case report from Brazil. Rev Soc Bras Med Trop. 2011;44(3):381–4.

[18] Gran K, Camara M, Diallo D, et al. Pediatric endomyocardial fibrosis secondary to Schistosoma intercalatum and S. haematobium co-infection: a case report from Guinea. Cardiol Young. 2011;21(5):515–20.

[19] Hotta T, Lima R, Oliveira F, et al. Trypanosoma cruzi-associated endomyocardial fibrosis in Brazil: case report. Rev Bras Cardiol. 2016;29(2):120–4.

[20] Martin W. Wuchereria bancrofti infection presenting as restrictive cardiomyopathy in Nigeria: a case report. Trop Cardiol. 2008;14(2):101–4.

[21] Mocumbi AO. Schistosomiasis-induced endomyocardial fibrosis in Mozambique: a pediatric case report. Heart Views. 2016;17(1):12–6.

[22] Onakpoya OH, El-Gohary Y, Abdallah M, et al. Endomyocardial fibrosis associated with Schistosoma mansoni infection: report of two Egyptian cases. J Trop Pediatr. 2010;56(5):341–5.

[23] Romero M, Silva R, Santos F, et al. Schistosoma mansoni-related endomyocardial fibrosis: case report from Brazil. Arq Bras Cardiol. 2022;119(4):765–70.

[24] Sarazin M, Tribouilloy C, Mahé I, et al. Endomyocardial fibrosis in a French patient with Schistosoma mansoni infection: case report and literature review. Eur Heart J. 2003;24(6):556–60.

[25] Mohamed Ayman H, El-Massry KM, Abdel-Aal F, et al. Schistosoma mansoni infection presenting with endomyocardial fibrosis: case series from Egypt. Am J Trop Med Hyg. 1995;52(6):519–24.

[26] Soarres FF, Almeida LM, Pinto LM, et al. Late-onset endomyocardial fibrosis in Brazil: a case report with left ventricular thrombosis. Arq Bras Cardiol. 2023;120(5):1020–5.

[27] Andrade JL, Souza AS, Reis VM, et al. Pathogenesis of schistosomal endomyocardial fibrosis: a histopathologic study. Am J Trop Med Hyg. 1996;55(2):125–32.8780448

[28] Siqueira RF, Rocha MO, Lopes AA, et al. Hepatosplenic schistosomiasis and cardiac involvement: a clinicopathological correlation. Rev Soc Bras Med Trop. 2001;34(1):25–30.11340493

[29] Mocumbi AO, Ferreira MB, Yacoub MH. Endomyocardial fibrosis: a form of restrictive cardiomyopathy. Heart. 2008;94(7):836–43.18552223

[30] Mocumbi AO. Endomyocardial fibrosis: a neglected tropical disease. Heart. 2012;98(21):1565–72.

[31] Mankad R, Bonnichsen C, Mankad S. Eosinophilic heart disease: a practical review. Clin Cardiol. 2016;39(9):547–54.

[32] Nutman TB. Lymphatic filariasis. Infect Dis Clin North Am. 2010;24(3):613–28.

[33] Marin-Neto JA, Cunha-Neto E, Maciel BC, et al. Pathogenesis of chronic Chagas heart disease. Circulation. 2007;115(9):1109–23. 17339569 10.1161/CIRCULATIONAHA.106.624296

[34] Graham BB, Langleben D, Simonneau G. Schistosomiasis-associated pulmonary arterial hypertension. Am J Respir Crit Care Med. 2010;182(2):123–32.

[35] Andrade ZA. Schistosomal pulmonary arterial hypertension: immunopathogenesis and treatment. Mem Inst Oswaldo Cruz. 2009;104(Suppl 1):139–46.

[36] Mehta SK, Shivaprasad C, Kumar V, et al. Pulmonary vascular lesions in schistosomiasis: histopathologic features. Am J Trop Med Hyg. 1998;59(3):388–92.9749630

[37] Fallon PG. Schistosome-induced immunopathology: lessons from animal models. Trends Parasitol. 2000;16(4):145–51.

[38] Brambilla DJ, den Hollander NS, Neumann DA, et al. Eosinophil-mediated cardiac injury in helminth infection. J Infect Dis. 1993;167(5):1254–8.

