# Supplementary material for: Parasitic infections and the development of endomyocardial fibrosis: systematic review of case reports and case series
Source: Trop Med Health. 2025 Aug 20;53:112. doi: 10.1186/s41182-025-00793-7 (PMC12366135; doi:10.1186/s41182-025-00793-7)
Supplement: Supplementary file 1 — Supplementary Material 1. [file 41182_2025_793_MOESM1_ESM.docx]

**Full Search Strategy**

Databases Searched

- PubMed
- Embase
- Scopus
- Google Scholar
- Virtual Health Library (VHL)
- ScienceDirect
- USC Libraries

Search Date

- All searches were conducted in February 2025.

Search Limits

- Language: English only (due to language proficiency limitations)
- Study Type: Case reports, case series
- No restrictions on publication date.
- No restrictions on geographical location.

Detailed Search Strategy (Initial to Refined)

- PubMed

Step 1: Initial Broad Search (overly general results found)

We began with a comprehensive search using the full MeSH hierarchy for parasitic diseases to capture all possible parasitic infections that could be related to endomyocardial fibrosis (EMF).

("Endomyocardial Fibrosis"[Mesh] OR "Endocardial Fibrosis"[Title/Abstract]) AND

("Parasitic Diseases"[Mesh] OR "Central Nervous System Parasitic Infections"[Mesh] OR "Central Nervous System Helminthiasis"[Mesh] OR "Central Nervous System Protozoal Infections"[Mesh] OR "Eye Infections, Parasitic"[Mesh] OR "Acanthamoeba Keratitis"[Mesh] OR "Onchocerciasis, Ocular"[Mesh] OR "Toxoplasmosis, Ocular"[Mesh] OR "Helminthiasis"[Mesh] OR "Cestode Infections"[Mesh] OR "Helminthiasis, Animal"[Mesh] OR "Nematode Infections"[Mesh] OR "Trematode Infections"[Mesh] OR "Intestinal Diseases, Parasitic"[Mesh] OR "Anisakiasis"[Mesh] OR "Balantidiasis"[Mesh] OR "Blastocystis Infections"[Mesh] OR "Cryptosporidiosis"[Mesh] OR "Dientamoebiasis"[Mesh] OR "Dysentery, Amebic"[Mesh] OR "Giardiasis"[Mesh] OR "Liver Diseases, Parasitic"[Mesh] OR "Echinococcosis, Hepatic"[Mesh] OR "Fascioliasis"[Mesh] OR "Liver Abscess, Amebic"[Mesh] OR "Lung Diseases, Parasitic"[Mesh] OR "Echinococcosis, Pulmonary"[Mesh] OR "Mesomycetozoea Infections"[Mesh] OR "Rhinosporidiosis"[Mesh] OR "Parasitemia"[Mesh] OR "Parasitic Diseases, Animal"[Mesh] OR "Protozoan Infections, Animal"[Mesh] OR "Pythiosis"[Mesh] OR "Pregnancy Complications, Parasitic"[Mesh] OR "Protozoan Infections"[Mesh] OR "Amebiasis"[Mesh] OR "Babesiosis"[Mesh] OR "Ciliophora Infections"[Mesh] OR "Coccidiosis"[Mesh] OR "Euglenozoa Infections"[Mesh] OR "Malaria"[Mesh] OR "Theileriasis"[Mesh] OR "Trichomonas Infections"[Mesh] OR "Skin Diseases, Parasitic"[Mesh] OR "Ectoparasitic Infestations"[Mesh] OR "Larva Migrans"[Mesh] OR "Leishmaniasis"[Mesh] OR "Onchocerciasis"[Mesh]) AND

("Case Reports"[Publication Type] OR "Case Series"[Title/Abstract])

Outcome: This search yielded 24 results in PubMed.

Step 2: Intermediate Narrowing

We the narrowed the search by focusing on clinically relevant parasitic diseases commonly associated with EMF based on existing literature

(("Endomyocardial Fibrosis"[Mesh] OR "Endomyocardial Fibrosis"[Title/Abstract] OR "Endocardial Fibrosis"[Title/Abstract]) AND

("Schistosoma mansoni"[Mesh] OR "Schistosoma haematobium"[Mesh] OR "Schistosoma mansoni"[Title/Abstract] OR "Schistosoma haematobium"[Title/Abstract] OR "Schistosomiasis"[Mesh] OR "Schistosomiasis"[Title/Abstract] OR

"Trypanosoma cruzi"[Mesh] OR "Trypanosoma cruzi"[Title/Abstract] OR "Chagas Disease"[Mesh] OR "Chagas Disease"[Title/Abstract] OR

"Wuchereria bancrofti"[Mesh] OR "Wuchereria bancrofti"[Title/Abstract] OR "Filariasis"[Mesh] OR "Filariasis"[Title/Abstract] OR

"Loa loa"[Mesh] OR "Loa loa"[Title/Abstract] OR "Loiasis"[Title/Abstract]) AND

("Case Reports"[Publication Type] OR "Case Series"[Title/Abstract] OR "Case Study"[Title/Abstract]))

Outcome: This search yielded 19 results

Step 3: Final Refined Search (Yielded Relevant Results)

We further refined the search to focus on specific parasites that have been directly implicated in EMF in prior reports.

("Endocardial Fibrosis"[Mesh] OR "Endomyocardial Fibrosis"[Mesh] OR "Endocardial Fibrosis"[Title/Abstract] OR "Endomyocardial Fibrosis"[Title/Abstract]) AND ("Parasitic Diseases"[Mesh] OR "Schistosomiasis"[Mesh] OR "Filariasis"[Mesh] OR "Trypanosomiasis"[Mesh] OR "Malaria"[Mesh] OR "Toxoplasmosis"[Mesh] OR "Schistosomiasis"[Title/Abstract] OR "Filariasis"[Title/Abstract] OR "Trypanosomiasis"[Title/Abstract] OR "Malaria"[Title/Abstract] OR "Toxoplasmosis"[Title/Abstract]) AND ("Case Reports"[Publication Type] OR "Case Series"[Publication Type] OR "Case Study"[Title/Abstract])

Outcome: This search yielded 34 relevant studies that provided usable data for the review.

- Embase, USC Libraries, VHL, and Scopus

(("Endomyocardial Fibrosis" OR "Endocardial Fibrosis") AND ("Parasitic Diseases" OR "Schistosomiasis" OR "Filariasis" OR "Trypanosomiasis" OR "Malaria" OR "Toxoplasmosis") AND ("Case Reports" OR "Case Series" OR "Case Study"))

- Google Scholar

(("Endomyocardial Fibrosis" OR "Endocardial Fibrosis") AND ("Parasitic Diseases" OR "Schistosomiasis" OR "Filariasis" OR "Trypanosomiasis" OR "Malaria" OR "Toxoplasmosis") AND ("Case Reports" OR "Case Series" OR "Case Study"))

(Note: Google Scholar does not support advanced Boolean operators in the same way as other databases; manual screening was conducted.)

- Supplementary Search Method

Backward snowballing: Reference lists of all included articles were reviewed to identify additional relevant studies.

- Screening Process

Titles and abstracts were independently screened by two authors.

Full texts were retrieved for studies meeting inclusion criteria.

Discrepancies were resolved through consensus discussion.

- Summary of Filters Applied

Language: English only

Study Type: Case reports, case series

No date restrictions

Human studies
