# Supplementary material for: Parasitic infections and the development of endomyocardial fibrosis: systematic review of case reports and case series
Source: Trop Med Health. 2025 Aug 20;53:112. doi: 10.1186/s41182-025-00793-7 (PMC12366135; doi:10.1186/s41182-025-00793-7)
Supplement: Supplementary file 2 — Supplementary Material 2. [file 41182_2025_793_MOESM2_ESM.docx]

**The quality assessment of the included studies was evaluated using the Joanna Briggs Institute (JBI) Critical Appraisal Checklists for case reports and case series.**

| **Study** | Patient’s demographics clearly  described?/Clear inclusion criteria | Patient's history clear and presented as timeline? /condition measured in a standard and reliable way? | Current clinical condition of the patient on  presentation clearly described?/valid methods used for identification of participants' conditions | Diagnostic, assessment methods and results clear? / Did the case series have consecutive inclusion of participants? | Intervention(s) or treatment procedure(s) clearly  described? /Did the case series have complete inclusion of  participants? | Post-intervention clinical condition clearly  described? / clear reporting of the demographics of  the participants in the study? | Were adverse events (harms) or unanticipated events identified and described? /Was there clear reporting of clinical information of the participants | Does the case report provide takeaway lessons? /Were the outcomes or follow up results of cases clearly reported? | Was there clear reporting of the presenting site(s)/clinic(s) demographic information? | Was statistical analysis appropriate? |
| --- | --- | --- | --- | --- | --- | --- | --- | --- | --- | --- |
| Assimeng et al, 2014 [3] | Yes | No | Yes | Yes | Yes | Yes | Yes | Yes | Not applicable | Not applicable |
| Mohamed Ayman et al, 1995 [4] | Yes | Yes | Yes | Yes | Yes | Yes | Yes | Yes | Yes | Yes |
| Bustinduy et al, 2014 [5] | Yes | No | Yes | Yes | Yes | Yes | Yes | Yes | Not applicable | Not applicable |
| Carneiro et al, 2011 [6] | Yes | No | Yes | Yes | Yes | Yes | Yes | Yes | Not applicable | Not applicable |
| Gran et al, 2011 [7] | Yes | No | No | Yes | Yes | Yes | Yes | No | Not applicable | Not applicable |
| Hotta et al, 2016 [8] | Yes | No | Yes | Yes | Yes | Yes | Yes | Yes | Not applicable | Not applicable |
| Martin, 2008 [9] | Yes | No | Yes | Yes | Yes | Yes | Yes | No | Not applicable | Not applicable |
| Mocumbi, 2016 [10] | Yes | No | Yes | Yes | Yes | Yes | Yes | Yes | Not applicable | Not applicable |
| Onakpoya et al, 2010 [11] | Yes | No | Yes | Yes | Yes | Yes | Yes | Yes | Not applicable | Not applicable |
| Romero et al, 2022 [12] | Yes | Yes | Yes | Yes | Yes | Yes | Yes | Yes | Not applicable | Not applicable |
| Sarazin et al, 2003 [13] | Yes | No | Yes | Yes | Yes | Yes | Yes | Yes | Not applicable | Not applicable |
| Soarres et al, 2023 [14] | Yes | No | Yes | Yes | Yes | Yes | Yes | Yes | Not applicable | Not applicable |
